# Mind the gap – Managing tuberculosis across the disease spectrum

**DOI:** 10.1016/j.ebiom.2022.103928

**Published:** 2022-03-23

**Authors:** Hanif Esmail, Liana Macpherson, Anna K. Coussens, Rein M.G.J. Houben

**Affiliations:** a MRC Clinical Trials Unit at University College London, UK; b Institute for Global Health, University College London, UK; c Wellcome Centre for Infectious Diseases Research in Africa, Institute of Infectious Disease and Molecular Medicine, University of Cape Town, South Africa; d Infectious Diseases and Immune Defense Division, The Walter and Eliza Hall Institute of Medical Research, Parkville, Australia; e Department of Medical Biology, University of Melbourne, Parkville, Australia; f TB Modelling Group, TB Centre, London School of Hygiene and Tropical Medicine, UK; g Department of Infectious Disease Epidemiology, London School of Hygiene and Tropical Medicine, UK

**Keywords:** Tuberculosis, Disease spectrum, Subclinical disease, Treatment, Diagnosis

## Abstract

We currently have a binomial approach to managing tuberculosis. Those with active disease, ideally confirmed microbiologically, are treated with a standard 6-month, multi-drug regimen and those with latent infection and no evidence of disease with shorter, one or two drug regimens. Clinicians frequently encounter patients that fall between these two management pathways with some but not all features of disease and this will occur more often with the increasing emphasis on chest X-ray-based systematic screening. The view of tuberculosis as a spectrum of disease states is being increasingly recognised and is leading to new diagnostic approaches for early disease. However, the 6-month regimen for treating disease was driven by the duration required to treat the most extensive forms of pulmonary TB and shorter durations appear sufficient for less extensive disease. It is time undertake clinical trials to better define the optimal treatment for tuberculosis across the disease spectrum.

## Introduction

The current treatment algorithm for tuberculosis (TB) has been shaped over the last 80 years by TB control priorities, trial designs and diagnostic approaches, routed in a paradigm that conceptualises TB into binary states of active disease and latent infection. We argue that TB is more accurately represented as a spectrum of disease states requiring new diagnostic and treatment approaches.

**Figure 1:**
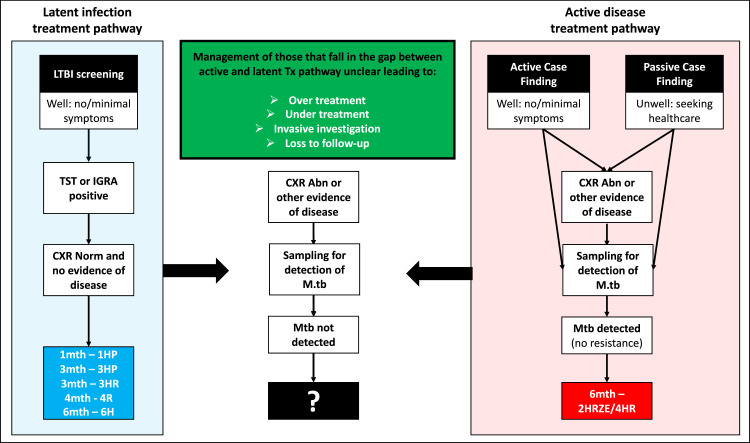
Shows an overview of the current management pathways for active and latent TB highlighting a group of patients that do not easily fit into either treatment algorithm and management is variable and practitioner dependent. In low income settings patients often get a trial of antibiotics leading to over use of antibiotics but ultimately the options are to provide empirical therapy with the standard 6-month regimen (over-treatment) or observation which can lead to loss to follow-up. Abn = abnormal, CXR = chest X-Ray, M.tb = *Mycobacterium tuberculosis.* R = Rifampicin, H = Isoniazid, Z = Pyrazinamide, E = Ethambutol, P = Rifapentine, TST = Tuberculin Skin Test, IGRA = Interferon Gamma Release Assay

**Figure 2:**
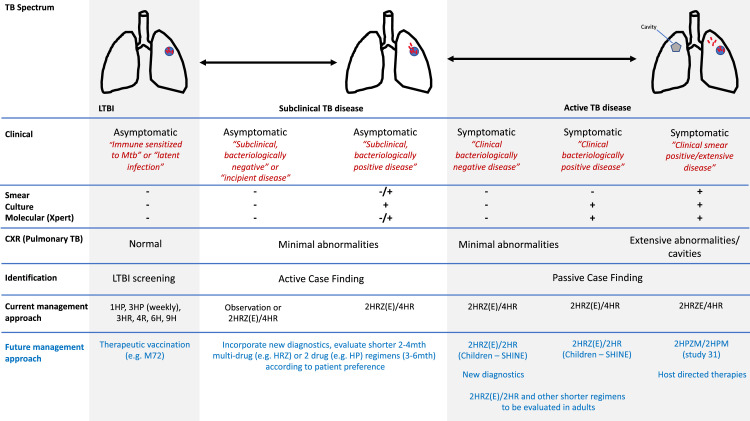
Shows the spectrum of disease from latent infection (determined by immune sensitization to Mtb) where Mtb is thought to be contained within granuloma though early stages of disease following Mtb escape resulting in infiltrative pathology and later stages resulting in more extensive tissue damage and cavitation in the context of pulmonary disease. The table the current diagnostic and treatment approaches across the spectrum. In blue future treatment strategies are outlined. LTBI = Latent tuberculosis infection, CXR = chest X-Ray*.* R = Rifampicin, H = Isoniazid, Z = Pyrazinamide, E = Ethambutol, P = Rifapentine, TST = Tuberculin Skin Test, IGRA = Interferon Gamma Release Assay.

Persons with latent TB (identified by demonstrating immune sensitization to *Mycobacterium tuberculosis* (Mtb)) are assumed to be infected with low numbers of organisms contained within granuloma, causing minimal pathology, with no symptoms or evidence of disease, where Mtb cannot be readily cultured from routine samples.^1^ Whereas those with active TB are assumed to have a large number of organisms, not contained by the immune response, causing manifest pathology and symptomatic presentation and where Mtb is typically culturable.^1^ In pulmonary TB, cavitation is recognized as a critical aspect of pathogenesis which occurs in a proportion, facilitating extracellular bacillary replication in an oxygen-rich niche. This contributes to significant increases in bacillary burden within respiratory secretions, facilitating microscopic visualisation (smear positive) as well as aiding transmission. Within this framework the overall treatment approach has been straightforward when not complicated by drug resistance (Figure 1); those considered to have active TB are recommended 6-month multidrug treatment (an intensive phase of 3/4 drugs [isoniazid (H) rifampicin (R), pyrazinamide (Z) +/- ethambutol (E)] for 2 months and a continuation phase of 2 drugs [HR] for 4 months with the aim of curing disease and preventing relapse.^2^ Whereas those considered to have latent TB receive 1 or 2 drugs (either a rifamycin or isoniazid or both) typically for 3 to 6 months with the aim of bacterial sterilisation to prevent the development of symptomatic disease.^3^ There have been benefits to this simplicity, particularly programmatically, but its limitations are increasingly apparent. Rather than being binary states, active and latent TB represent the extremes along a disease spectrum. Whilst the spectrum of disease states has long been recognised they are insufficiently reflected in our approach to management.^4^, ^5^, ^6^ (Figure 2)

## The development of the current management algorithm

### Active TB

In the pre-chemotherapy era, the inception of disease was well known to predate clinical presentation and the relapsing remitting nature of early disease was well understood.^7^ This resulted in widespread implementation of mass chest X-ray (CXR) screening in the mid 20th century to detect those with early disease.^8^^,^^9^ Patients presenting with advanced disease were recognised to have significantly worse outcomes.^9^^,^^10^ Hence, with the emergence of streptomycin chemotherapy in the 1940s, early trials in pulmonary TB focused on those with radiographically advanced disease not suitable for collapse therapy.^11^ Following the emergence of streptomycin resistance, the addition of two drugs (para-aminosalicylic acid (PAS) and isoniazid) was found to be necessary to prevent acquired resistance in those with extensive disease.^12^ With the development of rifampicin the British Medical Research Council (MRC) conducted a series of trials in the 1970’s and 1980’s to determine the optimal short course regimen (i.e. less than 12 months).^12^ These studies were initially conducted in those with smear positive (i.e. extensive/cavitary) pulmonary disease, establishing the 6-month short course regimen; isoniazid and rifampicin supplemented by pyrazinamide and streptomycin (S) for the first 2 months (2SHRZ/4HR) in the first instance (subsequently is was established that streptomycin was not needed as part of this regimen).^12^ Two year relapse rates were 1–3% with this 6-month regimen and reducing this to 4-months (2SHRZ/2HR) in the smear positive population increased relapse to 8–16%.^12^, ^13^, ^14^ Later trials in the 1980’s, conducted by the MRC and Hong Kong Chest Service (HKCS), in smear-negative pulmonary TB demonstrated that in those with lower burden of disease could be effectively treated with the shorter 4-month regimen (Table 1).^12^^,^^15^^,^^16^ Although this led to a change in some national TB guidelines (including temporarily for Hong Kong), few other trials were specifically conducted in this group resulting in limited evidence to support this reduced duration.^17^^,^^18^

**Table 1:** Summary of randomised control trials evaluating shorter than standard treatment duration (< 6 months) in participants with limited disease. Studies arranged by inclusion criteria from more extensive to less extensive disease as determined by smear, culture, radiology and symptoms.

Study, Country, Publication year	Population	Participants randomised	Microbiological investigations	CXR	TB symptoms	Randomised arms (total duration)	Main outcome	Refs.
**Johnson et al., Brazil, Philippines, Uganda,** **2009.**	Adults with pulmonary TB (HIV-ve)	394	Smear: positive or negative Culture: positive converted to negative by 2 months	Non-cavitary	Symptomatic	2HRZE/4HR (6mth) 2HRZE/2HR (4mth)	Outcome – Bacteriological or clinical relapse over 30 months **6mth: 1.6%** **4mth: 7%** (Study stopped early - 4 months inferior to 6 months)	^72^
**Hong Kong Chest Service, Hong Kong,** **1984.**	Adults with radiographic pulmonary TB	321	Smear: negative (5 samples) Culture: positive (≥1 of 5 samples)	Active pulmonary TB	The ‘majority’ had symptoms	3SHP/9S_2_H_2_ (12mth) 3SHRZ (3mth) 2SHRZ (2mth)	Outcome – Bacteriological or clinical/radiological relapse over 60 months **12mth: 5%** **3mth: 13%** **2mth: 32%**	^15^
**Hong Kong Chest Service, Hong Kong, 1989.**	Adults with radiographic pulmonary TB	502	Smear: negative (4 samples) Culture: positive (≥1 of 4 samples)	Active pulmonary TB	Symptomatic	6SHRZ_3_ (6mth) 4SHRZ (4mth) 4SHRZ_3_ (4mth)	Outcome – Bacteriological or clinical/radiological relapse over 60 months **6mth: 5%** **4mth (daily): 3%** **4mth (3x/week): 2%**	^16^
**Teo et al., Singapore, 2002**	Adults with radiographic pulmonary TB	113	Smear: negative (4 samples) Culture: positive (≥1 of 4 samples)	Active pulmonary TB	Symptomatic	2HRZ/4H_3_R_3_ (6mth) 2HRZ/2HR (4mth)	Outcome – Bacteriological relapse over 60 months **6mth: 2%** **4mth: 0%**	^18^
**SHINE Trial,** **South Africa, Uganda, Zambia, India, 2020.**	Children under 16 years with minimal TB: non-severe pulmonary, lymph node and pleural TB.	1,204	Smear: negative Culture: positive or negative	Minimal disease	Symptomatic	2HRZ (+/-E)/4HR (6mth) 2HRZ (+/-E)/2HR (4mth)	Outcome: TB treatment or failure, death, or on treatment LTFU over 72 weeks **6mth: 3%** **4mth: 3%**	^68^
**Hong Kong Chest Service, Hong Kong,** **1984.**	Adults with radiographic pulmonary TB (culture negative)	1,019	Smear: negative (5 samples) Culture: negative (5 samples)	Active pulmonary TB	The ‘majority’ had symptoms	3SHP/9S_2_H_2_ (12mth) 3SHRZ (3mth) 2SHRZ (2mth) Selective therapy if culture positive (SC)	Outcome – Bacteriological or clinical/radiological progression over 60 months **12mth: 2%** **3mth: 7%** **2mth: 11%** **SC: 57%**	^15^
**Hong Kong Chest Service, Hong Kong, 1989.**	Adults with radiographic pulmonary TB	1,118	Smear: negative (4 samples) Culture: negative (4 samples)	Active pulmonary TB	Symptomatic	4SHRZ_3_ (4mth) 3SHRZ (3mth) 3SHRZ_3_ (3mth)	Outcome – Bacteriological or clinical/radiological relapse over 60mths **4mth: 4%** **3mth (daily): 6%** **3mth (3x/week): 8%**	^16^
**Teo et al. Singapore, 2002**	Adults with radiographic pulmonary TB	201	Smear: negative (4 samples) Culture: negative (4 samples)	Active pulmonary TB	Symptomatic	2HRZ/2H_3_R_3_ (4mth) 2HRZ/2HR (4mth)	Outcome – Bacteriological relapse over 60 months **4mth (3x/week): 1%** **4mth: 0%**	^18^
**Cowie et al.,** **South Africa, 1979**	Gold mine employees, with apical lung lesions, positive TST	402	Smear: negative (3 samples) Culture: negative (2 samples)	New or enlarging apical lesions	Not reported	3RHZE (3mth) Selective therapy (SC)	Outcome – Bacteriological progression over 60 months **3mth: 14%** **SC: 58%**	^53^
**CORTIS trial, South Africa, 2016**	Adults (HIV-ve) Screened with blood biomarker (RISK11 TB transcriptional signature)	1,139	Smear: Not done Culture: Not done Xpert MTB/RIF: negative	Not done	Not reported	*Risk11 positive cases:* 3HP_7_ (3mth) Selective therapy (SC)	Outcome - Incidence of microbiologically confirmed pulmonary TB over 15 months **3mth: 1.94/100person years** **SC:2.09/100person years**	^60^

Very few studies explored optimal treatment duration in extra-pulmonary disease (EPTB).^12^^,^^19^ Aside from TB meningitis and spinal TB (which are treated with longer duration due to the consequence of relapse) the remainder of EPTB has been managed with the same regimen developed for smear-positive pulmonary TB.^2^ This despite the fact that the commonest manifestations of EPTB, pleural and lymph node TB, are typically paucibacillary and frequently culture negative.^20^ Similarly, management of paediatric disease followed that of adults despite marked differences in pathogenesis.^21^ Although disseminated disease does occur, particularly in infants <2 years, in pre-pubescent children disease is often minimal, frequently smear and culture negative and cavitation is rare.^22^

By the 1990s, following the declaration of TB as a global health emergency, the WHO DOTS strategy sought to simplify and standardise the approach to investigation and management of TB globally. It deemphasised the role of CXR, focused on symptom-based case detection and emphasised the importance of smear microscopy to identify those most contagious. Six-month multidrug therapy was recommended for all newly diagnosed cases of TB regardless of smear status, disease site (with the exception of TB meningitis) or population.^23^ Although diagnostics have now progressed with wider availability of culture and molecular diagnostics (such as Xpert MTB/RIF), a singular approach for treating active drug-sensitive TB still dominates national TB guidelines, in both low and high income settings.^24^, ^25^, ^26^

As a result, the management of all forms of active TB is driven by the duration and the number of drugs required for the most extensive and hardest to treat (i.e. cavitary, smear positive) form of pulmonary TB. However it is estimated that half of TB in adults and over 90% in children is smear negative.^27^ Further data from high income countries suggest that despite intensive investigation 15–20% of pulmonary TB in adults will be culture negative.^28^^,^^29^ The American national guidelines are notable as being one of the few to recognise a range of states across the disease spectrum and recommend 4 months treatment for culture negative disease, whilst acknowledging the limited evidence.^30^ However, reports suggest as few as 13% of those eligible for this shorter regimen receive it.^29^

### Latent TB

Therapy to prevent the development of active TB in those with latent TB emerged with the availability of isoniazid in 1952, as a cheap and relatively safe, well tolerated drug. It was initially assessed in children with primary disease, and subsequently evaluated for prevention of symptomatic TB in adults at high risk.^31^ Even in these early studies, CXR was used to ensure exclusion of active TB despite lack of symptoms. In those without HIV, evidence of immune sensitization through tuberculin skin testing (TST) or interferon gamma release assays (IGRA) was effective in distinguishing those with presumed latent infection that would benefit from preventive therapy. An initial duration of 12-months of isoniazid was reduced to 9- then 6-months. More recent studies evaluating addition of rifamycins have shown duration can be reduced to a least 3-months and potentially less.^32^ Current guidance for preventive therapy mandates the exclusion of active TB through confirming absence of symptoms and ideally use of CXR.^3^ The majority (typically over 95%) of those with a positive test for latent TB will not develop active TB if untreated. The number of patients with a positive latent TB test that we would need to treat in order to prevent one case of active TB is in the order of 30–100 depending on underlying epidemiological risk.^33^ Hence preventative treatments need to be acceptable to this generally well population who may not see significant individual benefit to treatment. For example, 2-month treatment with rifampicin and pyrazinamide although shown to be effective is no longer recommended due to adverse events.^34^

## The spectrum of TB disease

The current binomial TB management algorithm, defined by the extremes of TB disease and infection, is easy to implement provided patients meet diagnostic criteria (Figure 1). It broadly functions well in scenarios where patients seek healthcare because of symptoms and a microbiological diagnosis can be made or in settings where asymptomatic individuals are being screened for latent TB. However, patients are not only identified at these extremes of the disease spectrum as disease frequently evolves over months to years.

The failure of granulomatous control leads to the initial stage of disease which is manifest as infiltrative pathology.^35^, ^36^, ^37^, ^38^, ^39^ In the context of pulmonary disease this is evident as cellular infiltrate spreading within the bronchi, which can be visible radiographically.^35^^,^^40^^,^^41^ Disease trajectory from this stage can be variable and the drivers of this heterogeneity are poorly understood. Some take an accelerated course, while others may take a more chronic or undulating course and, in some instances, may self-heal without seeking medical evaluation.^4^^,^^6^ Symptoms such as cough fever and weight loss are mediated by a combination of factors including by cytokines generated by inflammatory cells and tissue damage at the site of disease, metabolic dysregulation and potentially by components of the Mtb cell wall itself.^42^, ^43^, ^44^ Hence, symptoms may not be evident until a certain threshold level of pathology is reached which may vary between individuals and may be affected by the infecting strain of Mtb.

A definitive diagnosis is currently dependent on microbiological isolation of Mtb generally within easily accessible samples. In the context of pulmonary disease, in lower resourced settings, this typically relies on a single spontaneously produced sputum sample sent for a molecular test such as Xpert MTB/RIF. However, in the early stages of the disease, shedding of bacilli into respiratory secretions may be limited or intermittent and cough may be absent or unproductive in nature resulting in poor quality samples. Sputum microbiology is therefore an insensitive surrogate for and poor correlate of true bacterial burden. While in more advanced disease symptoms and microbiologically positive samples will invariably accompany disease pathology, in states when disease pathology is less extensive this may not be the case: Patients may present with symptoms and evidence of TB pathology without microbiological positivity (clinically diagnosable, bacteriologically negative disease); or with microbiologically positive disease without symptoms (subclinical bacteriologically positive disease); or with evidence of disease radiographically without symptoms or positive microbiology (subclinical, bacteriologically negative disease)^15^^,^^45^ (Figure 2).

## The expanding gap in current management algorithms

Clinicians have long encountered patients who do not comfortably meet the criteria for either latent or active TB, unsatisfactorily falling between the two management algorithms (Figure 1). Patients frequently present with symptoms or radiographic evidence of TB disease where initial samples are microbiologically negative. In high-income settings they will undergo further investigations such as computed tomography followed by bronchoscopy or biopsy to obtaining specimens from closer to the disease site to help confirm the diagnosis microbiologically or histologically. In low- and middle-income settings, where disease burden is highest, such investigations are often either unavailable or unaffordable. The practice of trial of antibiotics with symptom observation to assist in distinguishing between bacterial pneumonia and TB is widespread and frequently recommended in guidelines. However, the evidence to support this to help diagnose TB is weak.^46^^,^^47^ The clinical decision is ultimately between empirical therapy developed for advanced disease, or observation with no therapy. This dichotomous choice, in which patients are either over-treated or under-treated, means either risking side effects or extended pathology. The scale of this problem is highlighted by the statistic that 43% of cases treated and reported to WHO as active TB in 2019 were not bacteriologically confirmed.^48^ In high income settings, where more sensitive detection and sampling methods are routine, this was reduced but remained at 16%.^48^

There is now recognition that millions of cases of TB fail to be diagnosed every year, an estimated 2.9 million were not notified in 2019.^48^ With an ambition to end the TB epidemic within a generation there is now renewed focus on targeted systematic screening for active TB in risk groups who have not sought care.^49^^,^^50^ Screening usually relies on initial evaluation of symptoms or CXR followed by confirmatory testing - typically bacteriological assessment of a single spontaneously produced sputum with molecular assays such as Xpert MTB/RIF.^51^ However, it has become increasingly apparent that the majority of undiagnosed cases in the community are more readily picked-up by CXR screening, than they are by symptoms.^45^ Hence the 2021 WHO screening guidelines now emphases the sensitivity of CXR as a screening tool for all populations and endorse the use of artificial intelligence (AI) Computer Aided Diagnostic (CAD) software for CXR interpretation for the first time.^50^^,^^52^^,^^53^ As the use of CAD technology and increasingly portable digital CXR become more feasible and affordable, CXR screening will become more accessible for communities where it would previously have been impossible.^54^^,^^55^ These factors mean that we will likely see an increase in the use of CXR as a tool for TB screening in many settings.

The aim of systematic screening is not only to find, but to successfully treat cases of active TB with the standard 6-month treatment regimen. However, those identified through screening are not seeking healthcare, and are often asymptomatic with less extensive disease hence treatment prolonged, intensive treatments may be expected to make patients feel worse rather than better. In addition, many will not have a productive cough and may be unable to produce a sputum sample for confirmatory diagnosis. In an observational study of 311,732 people undergoing community TB screening in Pakistan in 2018 and 2019, Habib et al. reported that 21,141 (6.8%) had abnormal CXR (above threshold CAD score) but of these only 7,677 (36.3%) had a sputum sample successfully collected and tested by Xpert MTB/RIF of which 581 were positive. However, of those positive 110 (19%) did not initiate treatment. The reasons for this were not known but the authors noted a gender difference with more women (24%) than men (17%) not initiating treatment, highlighting that cultural and contextual community factors may impact on uptake of treatment.^56^ The treatment preferences for otherwise healthy people screened for TB is an area for future research (Table 2)

**Table 2:** Research priorities for the management of TB across disease spectrum.

*RESEARCH PRIORITIES*	*POSSIBLE APPROACH*
**What are the preferences for treatment in people with subclinical disease (who may otherwise be well) and what are their reasons for refusal of treatment?**	Qualitative research and discrete choice experiments in different populations to understand views on pill burden, side effects, duration and efficacy and examine other cultural factors.
**What are the attitudes of healthcare workers and policy makers to moving away from a “one size fits all” strategy?**	Surveys to understand concerns, challenges and levels of enthusiasm for a more tailored approach.
**What are the economic consequences to moving away from a “one size fits all” model of TB treatment to a more nuanced algorithm,**	Health economic modelling studies
**What is the role of new diagnostics in identifying those likely to have bacteriologically negative TB disease and in what context are these tests best deployed?**	Observational cohorts evaluating new diagnostics with intensive sampling for disease confirmation or longitudinal follow-up for incident disease.
**What is the optimal therapy for the management of less extensive disease?**	Clinical trials conducted in different populations e.g. smear negative/culture positive disease, symptomatic culture negative disease, subclinical disease. Including pulmonary and extrapulmonary disease.
**Can treatment biomarkers inform duration?**	Evaluate putative biomarkers in clinical trial of observational study setting relating to treatment failure.

With increased CXR screening, we will identify more patients with abnormal radiographs suggestive of active TB but with negative confirmatory bacteriological tests. These individuals are at high risk of progressing to culture confirmed disease with rates of progression as high as 30% to 50% over 4–6 years.^15^^,^^57^^,^^58^ Thus, while some with abnormal CXR will represent a false positive for future disease progression (including some who may self-heal in the absence of treatment), a significant proportion will have early TB disease which could continue to evolve, with potential to intermittently shedding of bacilli into respiratory secretions contributing to transmission (Figure 2). Screening algorithms are often vague about management of this group in lower resource settings. This leads to a clinical judgement over whether or not to offer empirical 6-month treatment to those with limited disease radiographically and minimal symptoms. However the International Standards for Tuberculosis Care strongly recommend against treatment on the basis of radiographic changes alone.^47^ Despite this, in some settings where there has been an expansion of CXR screening, the proportion of patients getting empirical treatment has increased - leading to tensions within TB programs that are keen to encourage treatment with bacteriological confirmation.^59^^,^^60^

## New diagnostic strategies for early disease detection

The issue of diagnostics and treatment go hand-in-hand. The primary issue with current diagnostics used as confirmatory tests in active TB is that they are not sensitive during early stages of disease when samples are likely to be paucibacillary.

The next generation of more sensitive tests should ideally identify current clinically diagnosed cases that are bacteriologically negative, subclinical cases that are bacteriologically positive and subclinical cases that are bacteriologically negative (i.e. predictive of incident cases of bacteriologically positive disease) (Table 2).^61^ In recent years there have been advances in diagnostic development relevant to these early stages of disease (see Theron et al. review in this issue). Broadly these tests either detect a host response to disease caused by Mtb or detect components of the pathogen. Although these new diagnostics hold promise their role in clearly defining different points on the disease spectrum is yet to be fully elucidated.

There has been particular interest in blood RNA signatures as a new diagnostic modality especially since these now have the potential to be implemented at point of care with fast turnaround times.^62^ These tests identify a specific immune transcriptional response indicative of TB. The most parsimonious signatures which converge on genes involved in interferon signalling, distinguish those with active verses latent TB and meet WHO-defined sensitivity and specificity to predict those with latent TB who will progress to symptomatic disease in the next 6-months.^63^^,^^64^ However, in more pragmatic settings, leading candidate signatures performance is reduced due to interferon signature overlap with other common circulating viral infections.^65^^,^^66^

Immunological assays that characterise the activation state (e.g. through HLA-DR expression) of cytokine secreting Mtb-specific T cells may be more indicative of an immunological response to currently replicating bacteria i.e. where antigen is actively being presented to T cells. They can effectively distinguish those with active versus latent TB as well as those recently infected (defined by QuantiFERON conversion) and identify those that progress to incident TB from those that do not.^67^^,^^68^

On the pathogen side, non-sputum-based approaches show promise and highlight that alternate tests on alternate biospecimens could be confirmatory for TB. Face mask sampling for pulmonary disease captures exhaled and expectorated respiratory particles directly onto a sampling matrix attached to the inside of the mask over a collection period (e.g. 30–60 min) which is then processed for microbiological detection.^69^ In pilot studies this approach appears more sensitive than sputum sampling with face mask positivity proceeding sputum positivity.^69^ Urinary diagnostics for LAM detection although initially considered a diagnostic for disseminated multibacillary disease in advanced HIV are also becoming increasingly sensitive in HIV-uninfected persons although have yet to be demonstrated to be suitable for early disease states.^70^

Where tests individually may lack specificity alone, combining tests in a 2-step screening strategy could improve this. CXR is a sensitive screening tool for early disease detection, however, the majority of those with features suggestive of active TB or with scores above certain CAD threshold will have bacteriologically negative sputum. A proportion of these individuals will have true bacteriologically negative TB disease undetectable by current sputum diagnostics. Confirmation of TB could then be better distinguished by employing one of the novel tests listed above.

## Treatment strategies for early disease

Although there are some advantages to the simplicity of a single “one size fits all” treatment approach for those with TB disease particularly in lower resourced settings, there are also detrimental consequences. Systematic overtreatment of a significant proportion of individuals comes at the cost to both the health system and individual. Pill burden and side effects are frequently cited as key challenges with treatment by patients and lead to non-adherence.^71^ For patients with minimal or no symptoms (subclinical disease) the offer of 6-months multi-drug therapy may contribute to refusal (Table 2). Furthermore, the resources invested in health systems to ensure completion of treatment would be proportionately reduced by a reduction in treatment duration. It is possible that those with disease of more limited extent could be treated with fewer drugs, shorter durations, or both, but there have been limited trials to date (Table 1).

In many contexts those with early disease, particularly those that do not have symptoms and where CXR is not used to exclude active disease, may be misclassified and treated as having a latent infection. Until recently it has not been clear if latent regimens are sufficient to cure early disease. The CORTIS trial was the first to evaluate the efficacy of preventive therapy with weekly isoniazid and rifapentine (P) for 3 months (3HP) in adults not seeking healthcare who were screened for disease using a blood RNA biomarker (RISK-11).^65^ No CXR was conducted. Without treatment the prevalence of culture positive disease in those with a positive and negative RISK-11 signature was 4.1% and 0.78%, respectively. In those with a positive RISK-11 signature who were culture negative at baseline, TB incidence over 15 months was 2.09/100person years without treatment and 1.94/100person years in those provided 3HP (treatment efficacy 7.0% (95% CI –145 to 64·7). If the RISK11 signature identifies those in the early stages of disease the most likely explanation for the lack of efficacy of 3HP is failure to cure disease resulting in recrudescence (Table 1).

It is possible that longer durations of the two-drug regimen could be sufficient for more limited disease. In observational studies conducted in Arkansas the 1980s Dutt et al. showed that those with smear negative, culture positive pulmonary disease could be successfully treated with a 6-month regimen of rifampicin and isoniazid, with relapse rates in those that completed therapy of 2.4% over median follow-up of 45 months.^72^ While those that were smear and culture negative, completing a 4-month regimen of rifampicin and isoniazid had relapse rate of just 1.2% over a median of 44 months follow-up.^73^ However, isoniazid resistance rates in this population were low and these regimens have yet to be assessed in a randomised controlled trial.

Multi-drug regimens containing rifampicin, isoniazid and pyrazinamide may facilitate further shortening. In the MRC/HKCS trials conducted in the 1980s, symptomatic individuals with CXR changes suggestive of active TB and negative sputum smear had at least 4 sputum samples sent for culture. In those who were smear-negative with at least one culture-positive (drug-sensitive) sample, disease relapse rate in those treated with either 4-months or 6-months SHRZ based therapy, was 2% at 2 years. Whereas if the duration was reduced to 2- or 3- months relapse rates increased to 15% and 9%, respectively. In those with smear and culture negative TB, disease progression over 2 years was 4% with 2-months, 2, 3% with 3-months and 2% with 4-months of SHRZ based therapy. In comparison 40% of those without treatment progressed to culture positive TB over the same period.^15^^,^^16^ Although shortening the duration of a regimen is felt to be positive it is not clear that if this is done at the expense of pill burden and side effects whether it is desirable from a patient perspective and further work to determine patient preference in different scenarios is needed (Table 1).

Children often present with more minimal disease which is frequently culture negative but until recently have been managed similarly to adults. The recent SHINE trial was the first to address this issue randomising 1204 children with non-severe, smear-negative respiratory or extra-thoracic lymph node disease to either 4-months (2HRZ(E)/2HR) or 6-months (2HRZ(E)/4HR) treatment.^74^ Four months was found to be non-inferior to six months in terms of unfavourable outcome (treatment failure, TB recurrence, death of any cause and loss to follow-up).^75^ As a result, the WHO as of August 2021 recommends four instead of six months therapy for children and adolescents under 16 years with non-severe presumed drug sensitive TB.^76^ (Table 1)

An alternative approach to stratifying treatment according to disease extent at baseline would be to tailor duration according to response by use of treatment biomarkers (Table 2). There is interest in this strategy which could potentially enable an individualised approach, with shorter treatment duration for all those responding favourably and longer duration for those that were not irrespective of baseline features.^77^ However, there are likely to be a number of challenges. An in-treatment biomarker that adequately predicts relapse-free survival has yet to be identified and it is possible that a single marker may not be sufficiently accurate across all populations and co-morbidities and a multi-component biomarker may limit use outside trial settings. To date, a single trial has been conducted reducing treatment in response to a treatment biomarker. Johnson et al. conducted a study in which in TB patients with non-cavitary TB (although >60% smear positive) who had culture converted at 2 months were randomised to 4-months (2HRZE/2HR) vs 6-months (2HRZE/4HR) treatment. Significantly more patients relapsed in the 4-month arm, 7% vs 1.6% and the trial was stopped early.^78^ (Table 1)

In recent years a significant focus of trials in drug sensitive pulmonary TB has been to identify a 4-month regimen with a recently published study finally showing that a 4-month rifapentine and moxifloxacin containing regimen (2HPZM/2HPM) was non-inferior to the standard 6-month regimen (2HRZE/4HR) in those with mainly smear positive pulmonary disease (79% ≥ 1+).^79^ However, three earlier trials published in 2014 where a fluoroquinolone was substituted for ethambutol or isoniazid failed to show non-inferiority to the standard 6-month regimen in smear-positive pulmonary tuberculosis.^80^, ^81^, ^82^ Reanalysing this data Imperial et al. found that 4-month fluoroquinolone containing regimen was non-inferior in those with less extensive disease ≤1+ smear or non-cavitary disease leading to a recommendation that the next generation of trial move beyond the one size fits all to a risk stratified approach based on markers of disease burden and severity.^83^^,^^84^

## Outstanding questions

As categorization of TB disease moves from the current dichotomous model to being thought of as a spectrum of disease states, more work needs to be done not only to fully determine the best diagnostic and treatment strategies, as outlined, but also to understand how a more diverse management strategy would impact health systems as well as public and individual health (Table 2). This is likely to vary in differently resourced settings. Appropriately treating those with early stages of disease may benefit the individual through use of shorter, less toxic treatments, reducing post-diagnosis patient costs and prevention of sequelae associated with advanced disease. It may also benefit society through reducing transmission which ultimately may have a positive economic impact. However, there are consequences for health systems to altering current algorithms which may involve introduction of new tests or provision of a greater range of treatment regimens. Increasing costs may divert limited resources away from other aspect of TB or broader health services. In addition, increasing the complexity of management algorithms will impact of training required and potentially level of expertise need to appropriately implement care. Hence the broader health economic consequences of new approaches need to be carefully assessed.

Additionally, more work is needed to understand the acceptability of diagnosis and treatment for those with minimal disease who may otherwise feel well, to ensure a person-centred approach to care. Such individuals will need to be guided through the risks and benefits of treatment and health care providers will need to be mindful of and better understand stigmatisation associated diagnosis of TB disease especially in those that do not feel unwell.

## Conclusion

Over the last decade there has been increasing recognition that the binary distinction of active and latent TB for all its simplicity is not reflective of disease natural history. Increasingly our TB control priorities mean that through use of CXR screening we will identify greater numbers of individuals that are not comfortably managed within existing treatment algorithms. The diagnostics pipeline has responded to the need for better tests that can better detect paucibacillary disease, but we are stuck with a “one size fits all” approach to manage it. This was an important policy in the 1990s but no longer reflects the needs of the 2020s. Earlier stages of disease can clearly be treated with shorter, less intense regimens than standard 6-month multidrug approach, whilst regimens for latent TB may not be sufficient to cure early disease. There is therefore a clear need for well-designed, efficient clinical trials and broader research (Table 2) to determine the best approach to managing disease at different points along the spectrum, to provide better options for patients and the healthcare workers treating them.

## Search strategy

A search was conducted of the PubMed database using the search terms (TB or Tuberculosis) AND (culture-negative OR smear-negative OR "culture negative" OR "smear negative") restricted to clinical trial and randomised control trials

## Contributors

HE and LM wrote the first draft which further contributed to by AKC and RH. All authors approved final draft.

## Declaration of interests

Dr. Esmail reports grants from UK Medical Research Council, during the conduct of the study. Prof Houben reports a grant from the European Research Council (Action Number # 757699). Dr. Coussens reports a grant from the Australian Respiratory Council. Dr. Macpherson has nothing to disclose.
